# Association between Angiotensin I-Converting Enzyme Insertion/Deletion Polymorphism and Prognosis of Kidney Transplantation: A Meta-Analysis

**DOI:** 10.1371/journal.pone.0127320

**Published:** 2015-05-22

**Authors:** Zhengkai Huang, Bian Wu, Jun Tao, Zhijian Han, Xiao Yang, Lei Zhang, Xuzhong Liu, Zijie Wang, Ruoyun Tan, Min Gu, Changjun Yin

**Affiliations:** Department of Urology, The First Affiliated Hospital of Nanjing Medical University, Nanjing, 210029, China; Max-Delbrück Center for Molecular Medicine (MDC), GERMANY

## Abstract

**Purpose:**

Angiotensin I-converting enzyme (ACE) is crucial in the renin–angiotensin–aldosterone system. ACE insertion/deletion (I/D) polymorphism is a common genetic variation of this gene and is associated with several disease phenotypes. However, the results of published studies on the influence of this polymorphism on renal transplantation are inconsistent. Therefore, a meta-analysis was performed to evaluate the association between ACE I/D polymorphism and prognosis of kidney transplantation.

**Methods:**

A meta-analysis was performed based on 21 case–control studies from 12 publications (1497 cases and 2029 controls) and 10 studies with quantitative values from 5 publications (814 patients). Pooled odds ratios (ORs) and weighted mean differences (WMDs) with their corresponding 95% confidence intervals (CIs) were used to estimate associations.

**Results:**

ACE I/D polymorphism was found to be associated with acute rejection (AR) in genotypes DD+ID versus II (OR = 1.62, 95% CI = 1.14–2.29) and with serum creatinine concentration after renal transplantation in genotypes DD versus ID (WMD = 13.12, 95% CI = 8.09–18.16). Stratified analysis revealed that recipients transplanted within a year had higher serum creatinine concentrations in the DD versus ID model. No significant association was found between hypertension and ACE I/D polymorphism.

**Conclusion:**

ACE I/D polymorphism is associated with AR and allograft function after kidney transplantation.

## Introduction

Renal transplantation is considered the best therapeutic approach for patients with end-stage renal disease because it significantly improves the quality of life of patients [1]. Graft function is influenced by various immunologic and non-immunologic factors, such as primary disease, immunosuppressive regimen, metabolic and cardiovascular conditions, episodes of acute rejection (AR) or chronic rejection (CR), and donor and recipient ages [2, 3]. Genetic factors play important roles in allografts by affecting blood pressure regulation, vascular proliferation and inflammatory responses, such as thrombosis, fibrosis or chemotaxis [4–6]. Under the same environment, recipients with different genotypes in a specific gene usually have different outcomes after transplantation. Based on data from the large number of kidney transplantations performed worldwide and data on long-term survival after the operation, researchers have established several associations between specific genotypes and graft outcome.

The renin–angiotensin–aldosterone system (RAS), which regulates homeostasis, vascular tone, blood pressure and salt balance, plays important roles in renal and cardiovascular physiologies [7–10]. Angiotensin I-converting enzyme (ACE) is the key enzyme that influences RAS activity by converting angiotensin I into vasoactive and aldosterone-stimulating peptide angiotensin II [11]. Poor renal transplant function and low survival of renal allografts are associated with RAS over-activation [5, 12]. Thus, ACE is likely to be an important determinant of prognosis after kidney transplantation.

The gene encoding ACE is located in chromosome 17, and individual patients may exhibit presence (I allele, insertion) or absence (D allele, deletion) of a 287-base pair Alu repeat sequence in intron 16 of this gene [13]. Thus, patients can be of three genotypes with regard to ACE, namely, II, ID and DD. Homozygotes of the D allele express higher levels of ACE compared with the other genotypes [14].

Many studies have investigated the association between ACE I/D polymorphism and renal transplantation, but their results are inconsistent. Considering the decentralised nature of patient data and inconsistent conclusions amongst the reported articles, it has been extremely difficult to assess the validity of the proposed theories of associations. Furthermore, a thorough study of the prognostic aspect of kidney transplantation and ACE I/D polymorphism remains lacking to date. In this study, we performed a meta-analysis to investigate whether ACE allele genotype has any influence on the prognosis of kidney transplantation.

## Materials and Methods

### Literature Search

**Fig 1:**
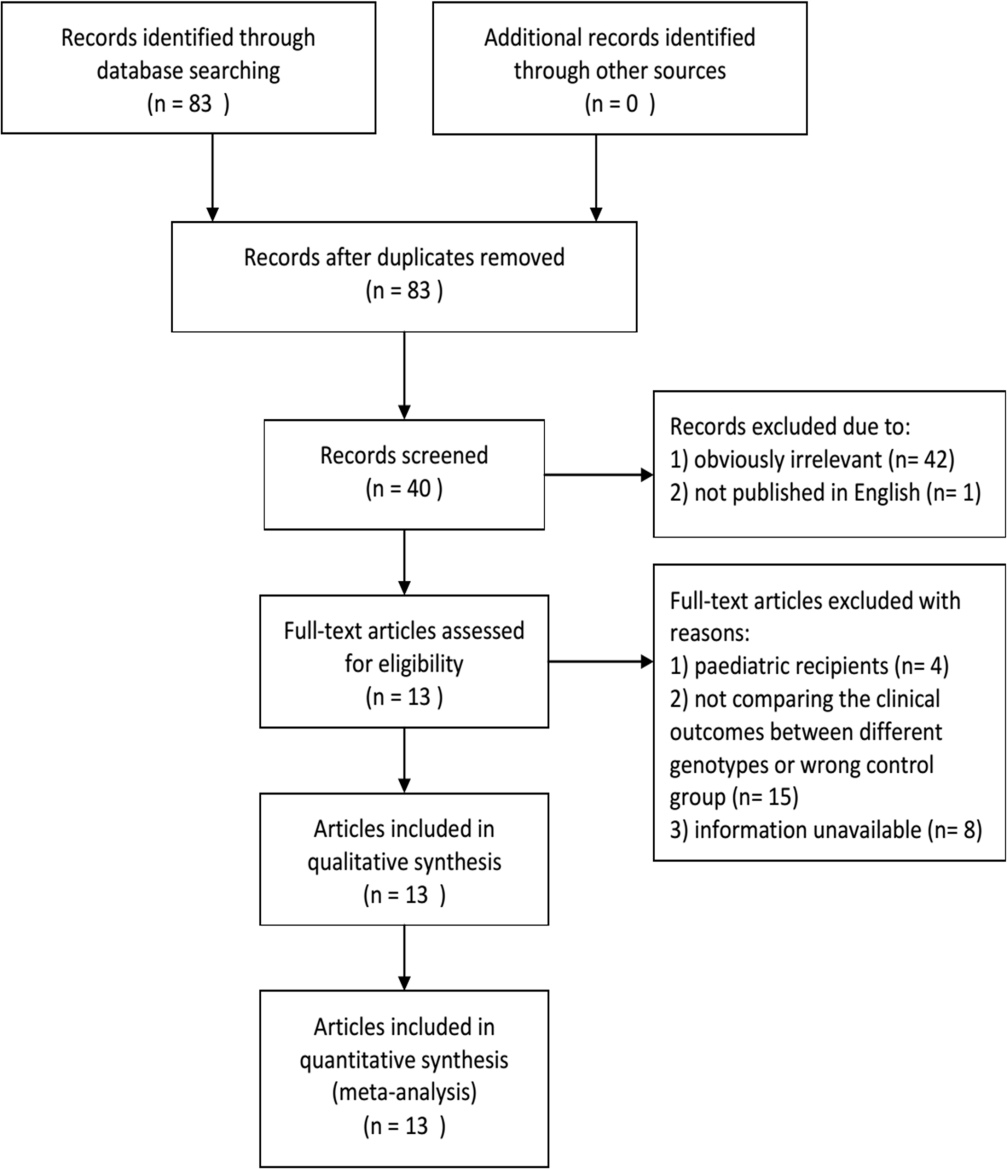
Flow diagram. Flow chart of search and selection.

To obtain relevant literature, a systematic search was performed using the PubMed and Embase (1966 to March 2014) databases with the following keywords: (‘ACE’ or ‘angiotensin converting enzyme’), (‘polymorphism’), (‘insertion/deletion’, ‘deletion/insertion’, ‘I/D’ or ‘D/I’), (‘transplant’ or ‘transplantation’), (‘allograft’) and (‘kidney’ or ‘renal’). Only articles written in English were included in the search. All eligible publications on the association between kidney transplantation and ACE I/D polymorphisms were searched. The references listed in the articles found were used as sources of supplementary information. Fig 1 shows the flow diagram depicting the overall strategy for inclusion of published literature in this study.

### Inclusion and Exclusion Criteria

All eligible studies on the association between ACE I/D polymorphisms and kidney transplantation were considered for inclusion using the following criteria: (1) studies must be published in English; (2) studies must be a case–control or cohort study of renal transplant recipients; and (3) studies should have provided data on the ACE genotypes II, ID and DD. In particular, detailed numbers of genotypes must have been provided to calculate the odds ratio (OR) or weighted mean difference (WMD) and the corresponding 95% confidence intervals (95% CIs). The exclusion criteria were as follows: (1) studies not evaluating the correlation between ACE I/D polymorphism and kidney transplantation; (2) animal or cellular studies; (3) reviews, comments, meta-analysis or abstracts; (4) studies that lack a control group; and (5) paediatric studies. For studies with overlapping case series, the largest sample sizes were considered.

### Data Extraction

Information from the publications that met the above criteria was carefully extracted by two independent investigators (Zhengkai Huang and Bian Wu). A third investigator (Jun Tao) subsequently reviewed the results. Discussions were personally conducted to reach a consensus in case of a disagreement. The following items were extracted from each article: first author’s name, publication year, ethnicity, genotyping method, source of controls, numbers of case and control patients in each genotype, time points, prognostic indicator and Hardy–Weinberg equilibrium (HWE) of genotype distribution. Ethnicity was categorised according to research area. When the data were not directly provided, we calculated the number based on its percentage and total amount presented by the articles. Only information from one replicate randomly selected among repeated measurements was used. In addition, serum creatinine level and blood pressure were converted to identical units of measurement for ease of comparison.

### Statistical Analysis

Pooled OR or WMD and the corresponding 95% CIs were calculated to estimate the strength of association between ACE I/D polymorphism and prognosis of kidney transplantation. A random- or fixed-effects model of rejection events, serum creatinine concentrations and hypertension was used in this meta-analysis. ORs were accurately measured for DD versus ID+II, DD+ID versus II, DD versus II, ID versus II and D allele versus I allele. Meanwhile, WMDs were determined for DD versus II, DD versus ID and ID versus II. In addition, subgroup stratification analyses were performed according to ethnicity or time points after transplantation to explore the association of ACE I/D polymorphism with transplantation outcomes.

*Q* and *I* ^*2*^ statistic tests were used to evaluate heterogeneity. *I* ^*2*^ > 50% indicates heterogeneity [15]. When *I* ^*2*^ > 50%, the random-effects model was used; otherwise, the fixed-effects model was utilised. The allele frequencies of the ACE I/D polymorphism from each included article were determined through allele counting. Furthermore, HWE was examined using Chi-square tests. To evaluate publication bias, Begg’s test and funnel plot were used [16, 17]. In addition, sensitivity analysis of the positive results was performed. All statistical analyses were performed using STATA 10.0 software (StataCorp, College Station, TX, USA).

## Results

### Study Characteristics

Using the search strategies mentioned above, 83 articles were initially identified. Of these, 40 articles were selected for full-text review and 43 were excluded according to the inclusion/exclusion criteria. A total of 27 of the 40 articles were excluded after full-text review because of paediatric recipients or unavailable information. The total baseline characteristics of the included studies are shown in Tables 1 and 2. All included articles were published from 1997 to 2013. Three studies investigated CR [1, 18, 19], whereas eight studies focused on AR [1, 18–24]. Ten studies from five publications included serum creatinine concentration [1, 19–21, 25]. Ten studies from eight publications considered hypertension [18, 19, 21, 24, 26–29]. The genotype and allele distributions of the included studies about rejections and hypertension are presented in Table 1. Meanwhile, the genotypes and serum creatinine levels of the included studies are shown in Table 2. The results of HWE test for ACE I/D genotype distribution in the control population are also presented in Table 1. Only one of the included studies was not under HWE [23].

**Table 1:** Main characteristics of these studies included in this meta-analysis (Genotype and allele distributions of rejections and hypertension).

First Author	Year	Ethnicity	Genotyping Method	SC	Time Points post-transplantation (months)	Genotyping Cases			Controls			Variable Type	HWE
						II	ID	DD	II	ID	DD		
Beige, J.[18]	1997	Caucasian	PCR-RFLP	HB	/	9	18	7	42	131	62	CR	0.059
Kabat-Koperska, J.[1]	2005	Caucasian	PCR-RFLP	HB	/	2	3	5	18	35	31	CR	0.18
Amorim, C. E.[19]	2013	Brazilian*	PCR-RFLP	HB	/	5	28	50	30	64	38	CR	0.759
Beige, J.[18]	1997	Caucasian	PCR-RFLP	HB	/	5	27	7	46	122	62	AR	0.317
Broekroelofs, J.[20]	1998	Caucasian	PCR-RFLP	HB	12	33	77	39	58	110	50	AR	0.876
Viklicky, O.[21]	2001	Caucasian	PCR-RFLP	HB	/	2	2	5	7	8	6	AR	0.279
Yildiz, A.[24]	2002	Caucasian	PCR-RFLP	HB	12	16		13	66		46	AR	/
Akcay, A.[22]	2004	Caucasian	PCR-RFLP	HB	/	21		27	36		41	AR	/
Kabat-Koperska, J.[1]	2005	Caucasian	PCR-RFLP	HB	/	6	10	7	14	28	29	AR	0.142
Zhang, G.[23]	2007	Asian	PCR-RFLP	HB	/	3	9	7	55	108	24	AR	0.01
Amorim, C. E.[19]	2013	Brazilian*	PCR-RFLP	HB	/	5	24	52	30	68	36	AR	0.844
Beige, J.[18]	1997	Caucasian	PCR-RFLP	HB	30	27	91	43	24	58	26	Hypertension	0.439
Hernandez, D.[28]	1997	Caucasian	PCR-RFLP	HB	6	19		16	3		0	Hypertension	/
Hernandez, D.[28]	1997	Caucasian	PCR-RFLP	HB	12	18		15	4		1	Hypertension	/
Beige, J.[26]	1998	Caucasian	PCR-RFLP	HB	>120	12	27	19	6	14	8	Hypertension	0.978
Beige, J.[26]	1998	Caucasian	PCR-RFLP	HB	<36	7	42	17	5	11	5	Hypertension	0.827
Viklicky, O.[21]	2001	Caucasian	PCR-RFLP	HB	>180	6	7	5	3	3	6	Hypertension	0.106
Basset el, E. A.[29]	2002	Caucasian	PCR-RFLP	HB	60	30	91	48	9	32	25	Hypertension	0.806
Yildiz, A.[24]	2002	Caucasian	PCR-RFLP	HB	12	50		31	32		28	Hypertension	/
Amorim, C. E.[19]	2013	Brazilian*	PCR-RFLP	HB	/	6	13	10	29	79	78	Hypertension	0.234
Chudek, J.[27]	2013	Caucasian	PCR-RFLP	HB	/	68	178	77	7	17	15	Hypertension	0.574

HB: Hospital-based Study; SC: source of controls; HWE: Hardy Weinberg Equilibrium; CR: Chronic rejection; AR: Acute rejection episodes. Case group: patients occurred acute rejection or chronic rejection or hypertension; Control group: not occurred acute rejection or chronic rejection or hypertension.

* term Brazilian represented Brazilian population.

**Table 2:** Main characteristics of these studies included in this meta-analysis (Genotypes and serum creatinine levels).

First Author	Year	Ethnicity	Genotyping Method	SC	Time Points post-transplantation (months)	II			ID			DD		
						N	Mean	SD	N	Mean	SD	N	Mean	SD
Broekroelofs, J.[20]	1998	Caucasian	PCR-RFLP	HB	12	91	170.0	85.0	187	158.0	59.0	89	171.0	87.0
Viklicky, O.[21]	2001	Caucasian	PCR-RFLP	HB	>180	9	167.9	89.6	10	139.4	41.9	11	116.8	23.0
Kabat-Koperska, J.[1]	2005	Caucasian	PCR-RFLP	HB	>10	20	192.7	60.1	38	160.0	56.6	36	163.6	66.3
Kabat-Koperska, J.[1]	2005	Caucasian	PCR-RFLP	HB	>10	20	184.8	65.4	38	152.9	42.4	36	160.9	60.1
Kabat-Koperska, J.[1]	2005	Caucasian	PCR-RFLP	HB	>10	20	192.7	83.1	38	160.0	63.7	36	160.0	67.2
Argani, H[25]	2007	Asian	PCR-RFLP	HB	/	19	104.3	16.8	54	113.2	22.1	35	123.8	25.6
Amorim, C. E.[19]	2013	Brazilian*	PCR-RFLP	HB	7 (days)	35	141.5	53.0	92	185.7	132.6	88	203.3	123.8
Amorim, C. E.[19]	2013	Brazilian*	PCR-RFLP	HB	1	35	123.8	26.5	92	132.6	44.2	88	150.3	53.0
Amorim, C. E.[19]	2013	Brazilian*	PCR-RFLP	HB	6	35	123.8	26.5	92	123.8	35.4	88	141.5	35.4
Amorim, C. E.[19]	2013	Brazilian*	PCR-RFLP	HB	12	35	123.8	26.5	92	123.8	35.4	88	141.5	44.2

HB: Hospital-based Study; SC: source of controls.

* term Brazilian represented Brazilian population.

### Genotype and Rejection Episodes

**Fig 2:**
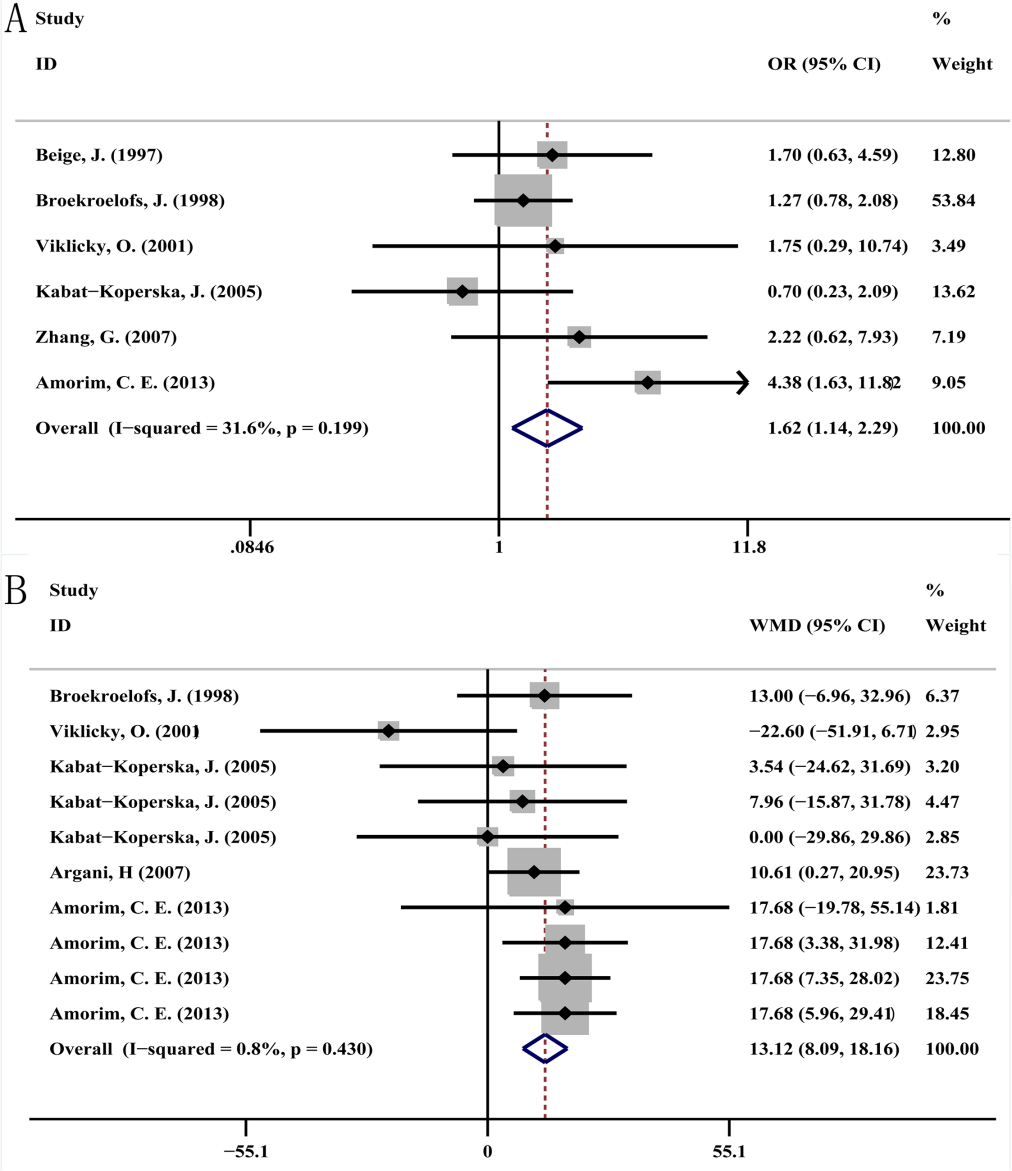
Forest plot of AR and serum creatinine concentration. (A) Forest plot of AR associated with ACE I/D polymorphism (for DD+ID versus II) among all studies. OR = 1.62, 95% CI = 1.14–2.29, I^2^ = 31.6, fixed-effects model. (B) Forest plot of serum creatinine concentration associated with ACE I/D polymorphism (for DD versus ID) among all included studies. WMD = 13.12, 95% CI = 8.09–18.16, I^2^ = 0.8, fixed-effects model. AR: acute rejection; OR: odds ratio; WMD: weighted mean difference.

In this meta-analysis, a significant association was detected between ACE I/D polymorphism and AR. The genotype model DD+ID versus II (dominant model) showed a significant difference in AR (OR = 1.62, 95% CI = 1.14–2.29, fixed-effects model). However, the other genotypes did not show any association: DD versus II, OR = 2.11, 95% CI = 0.91–4.00; ID versus II, OR = 1.39, 95% CI = 0.96–2.02; DD versus ID+II (recessive model), OR = 1.52, 95% CI = 0.86–2.70; D allele versus I allele, OR = 1.50, 95% CI = 0.92–2.45 (Table 3, Fig 2). In addition, no significant association between ACE I/D polymorphisms and CR was found amongst the five genetic models. When these rejection episodes were compared, no difference was observed amongst the five genetic models (Table 3). Subgroup analysis revealed high rejection risk among Brazilian population with the D allele genotype, but not among Caucasians (Table 3).

**Table 3:** Summary OR of ACE I/D genotype and rejection of all pooled studies.

Variables	n	DD versus II		ID versus II		DD+ID vs. II (dominant)		DD vs. ID+II (recessive)		D allele vs. I allele	
		OR (95% CI)	*I* ^2^ (%)	OR (95% CI)	*I* ^2^ (%)	OR (95% CI)	*I* ^2^ (%)	OR (95% CI)	*I* ^2^ (%)	OR (95% CI)	*I* ^2^ (%)
Overall	11	2.04 (0.98–4.24)	72.6 ^a^	1.33 (0.97–1.83)	0.0	1.59 (0.97–2.61)	53.3 ^a^	1.58 (0.98–2.57)	74.7 ^a^	1.49 (0.99–2.26)	81.1 ^a^
Type											
AR	8	2.11 (0.91–4.00)	69.1 ^a^	1.39 (0.96–2.02)	0.0	**1.62 (1.14–2.29)**	**31.6**	1.52 (0.86–2.70)	75.3 ^a^	1.50 (0.92–2.45)	80.3 ^a^
CR	3	1.85 (0.30–11.46)	84.6 ^a^	1.13 (0.41–3.08)	53.7 ^a^	1.46 (0.36–5.96)	79.6 ^a^	1.73 (0.56–5.30)	79.0 ^a^	1.46 (0.55–3.90)	87.9 ^a^
Ethnicity											
Caucasian	8	1.08 (0.71–1.63)	0.0	1.12 (0.77–1.62)	0.0	1.12 (0.79–1.58)	0.0	1.02 (0.77–1.35)	0.0	1.03 (0.84–1.26)	7.7
Brazilian*	2	**8.27 (3.97–17.22)**	**0.0**	**2.36 (1.13–4.97)**	**0.0**	**4.49 (2.22–9,04)**	**0.0**	**4.26 (2.82–6.45)**	**0.0**	**3.20 (2.34–4.37)**	**0.0**
Others	1	/	/	/	/	/	/	/	/	/	/

^a^ Random effects estimate

* term Brazilian represented Brazilian population.

### Genotype and Serum Creatinine

Ten studies measured serum creatinine in the three ACE I/D genotypes after renal transplantation. Regardless of the time points after transplantation, a significant difference in serum creatinine level was found in the DD versus ID model (WMD = 13.12, 95% CI = 8.09–18.16). However, this was not found in the DD versus II and ID versus II models (WMD = 9.18, 95% CI = −3.51–21.87 and WMD = −1.93, 95% CI = −11.75–7.89, respectively; Table 4, Fig 2). Stratified analysis between polymorphisms and time points after transplantation was performed on the above models. A significant difference in the same model was detected only in the group that had undergone transplantation for less than a year (DD versus ID), WMD = 14.81, 95% CI = 7.58–22.05. Furthermore, subgroup analyses revealed the II and DD genotypes to be risk factors among Caucasians and Brazilian population, respectively (Table 4).

**Table 4:** Summary WMD of the ACE I/D genotype and serum creatinine level.

Variables	n	DD versus II		DD versus ID		ID versus II	
		WMD (95% CI)	*I* ^2^ (%)	WMD (95% CI)	*I* ^2^ (%)	WMD (95% CI)	*I* ^2^ (%)
Overall	10	9.18 (-3.51–21.87)	73.4 ^a^	**13.12 (8.09–18.16)**	**0.8**	-1.93 (-11.75–7.89)	63.0 ^a^
Time							
>1year	3	1.72 (-24.65–28.10)	65.1 ^a^	6.46 (-13.40–26.33)	68.1 ^a^	-3.65 (-13.35–6.05)	0.0
<1year	6	7.22 (-14.86–29.30)	81.7 ^a^	**14.81 (7.58–22.05)**	**0.0**	-4.48 (-22.34–13.39)	74.1 ^a^
unknown	1	/	/	/	/	/	/
Ethnicity							
Caucasian	5	**-18.41 (−34.03- −2.80)**	**7.8**	3.17 (-8.13–14.48)	2.5	**-22.28 (−35.82- −8.74)**	**0.0**
Brazilian*	4	**25.11 (12.91–37.30)**	**60.7** ^**a**^	**17.68 (10.98–24.39)**	**0.0**	6.79 (-4.50–18.09)	59.8 ^a^
Others	1	/	/	/	/	/	/

^a^ Random effects estimate

* term Brazilian represented Brazilian population.

### Genotype and Blood Pressure

The results obtained from the meta-analysis of ACE I/D polymorphism and blood pressure are summarised in Table 5. However, the overall and stratified analyses showed no statistical difference among the five genetic models. The ethnicity and time points after renal transplantation were considered in the stratified analyses.

**Table 5:** Summary OR of the ACE I/D genotype and hypertension.

Variables	n	DD versus II		ID versus II		DD+ID vs. II (dominant)		DD vs. ID+II (recessive)		D allele vs. I allele	
		OR (95% CI)	*I* ^2^ (%)	OR (95% CI)	*I* ^2^ (%)	OR (95% CI)	*I* ^2^ (%)	OR (95% CI)	*I* ^2^ (%)	OR (95% CI)	*I* ^2^ (%)
Overall	10	0.87 (0.59–1.28)	11.8	1.15 (0.80–1.65)	0.0	1.03 (0.73–1.45)	0.0	0.82 (0.63–1.07)	0.6	0.91 (0.76–1.11)	14.4
Ethnicity											
Caucasian	9	0.91 (0.60–1.38)	21.6	1.20 (0.82–1.77)	0.0	1.08 (0.75–1.55)	0.0	0.83 (0.63–1.10)	11.1	0.94 (0.76–1.15)	24.2
Non-Caucasian	1	/	/	/	/	/	/	/	/	/	/
Time											
>3year	3	0.67 (0.34–1.31)	0.0	0.92 (0.48–1.75)	0.0	0.80 (0.44–1.46)	0.0	0.72 (0.45–1.17)	0.0	0.81 (0.58–1.12)	0.0
<3year	5	1.61 (0.83–3.12)	0.0	1.57 (0.88–2.80)	0.0	1.58 (0.91–2.75)	0.0	1.06 (0.72–1.56)	0.0	1.21 (0.89–1.65)	0.0
Unknown	2	0.56 (0.27–1.16)	0.0	0.95 (0.47–1.91)	0.0	0.77 (0.40–1.49)	0.0	0.59 (0.35–1.01)	0.0	0.72 (0.50–1.04)	0.0

^a^ Random effects estimate

### Sensitivity analysis

**Fig 3:**
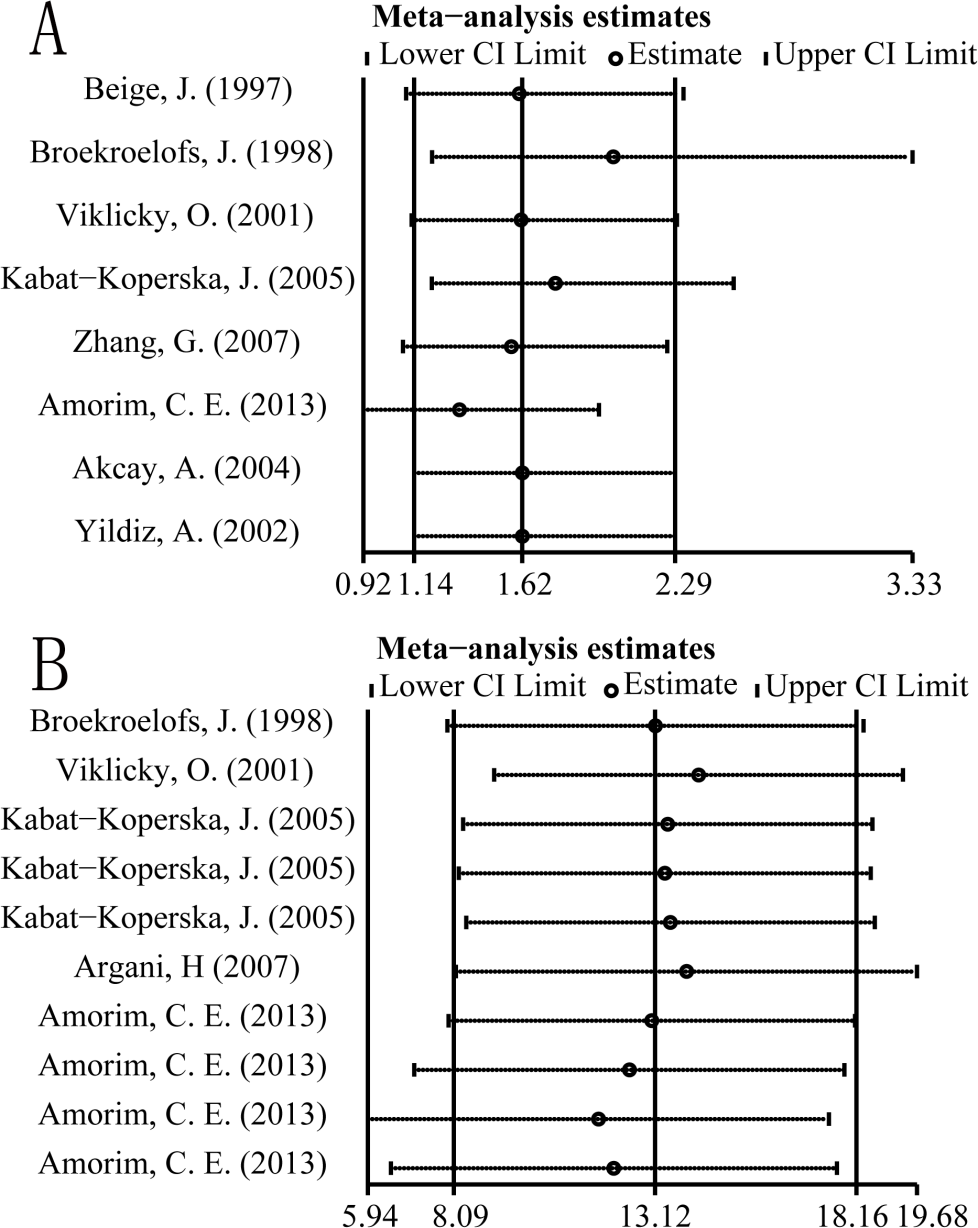
Influential analysis. (A) Influence of each study in the DD+ID versus II model on summary OR. No significant difference was found. (B) Influence of each study in the DD versus ID model on the summary WMD. No significant difference was found.

Sensitivity analysis of the positive results was performed to determine the influence of each study on the pooled ORs or WMDs by sequentially removing one study. No significant change was found, indicating that the results were reliable (Fig 3).

### Publication Bias

**Fig 4:**
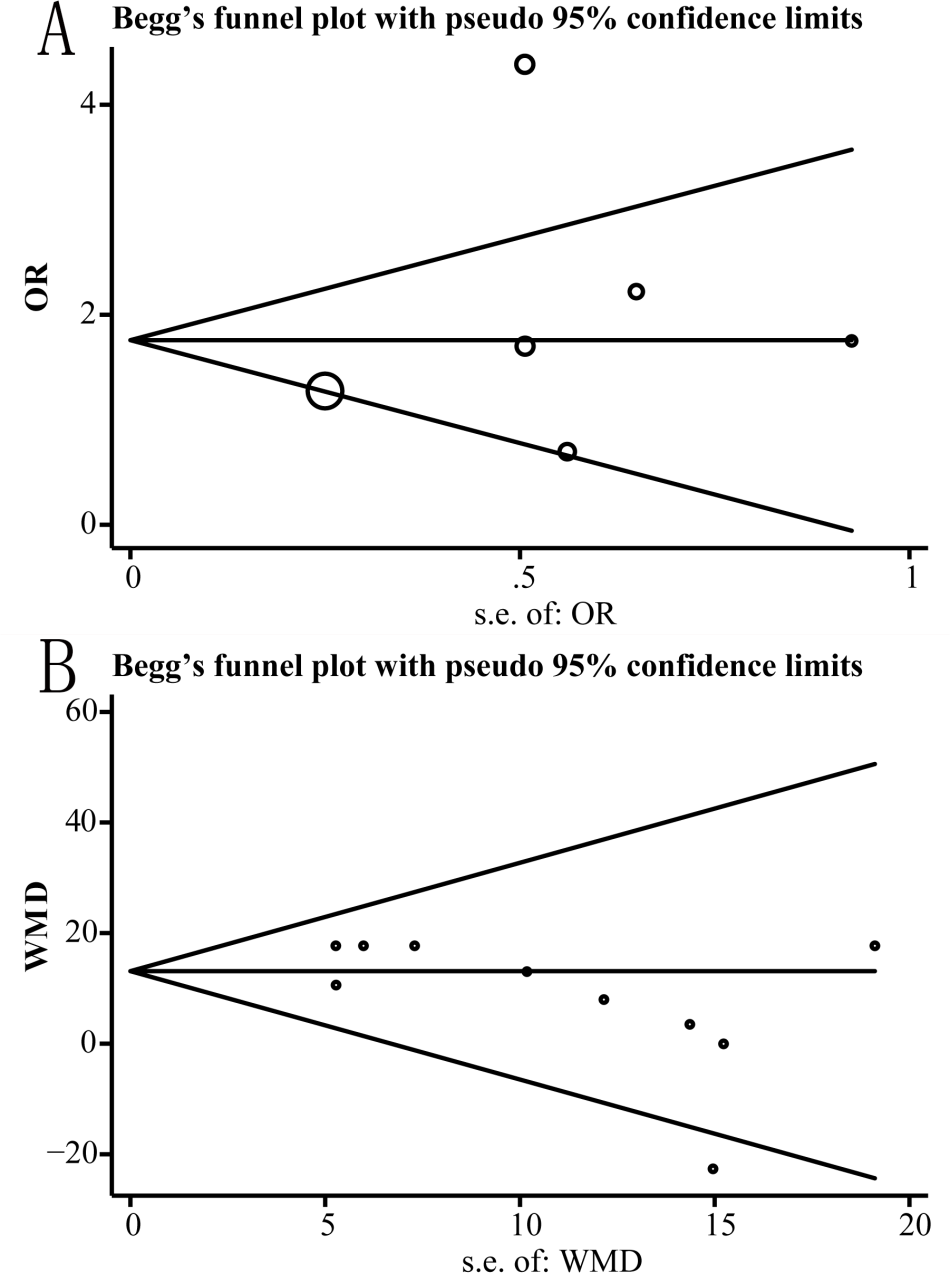
Begg’s funnel plot. (A) Begg’s funnel plot of association between ACE I/D polymorphism and acute rejection (AR) (DD+ID versus II). P = 0.521. (B) Begg’s funnel plot of association between ACE I/D polymorphism and serum creatinine concentration (DD versus ID). P = 0.073.

Publication bias was evaluated using Begg’s test and funnel plot. The results of the funnel plot analyses of groups AR and serum creatinine are shown in Fig 4 (group AR: DD+ID versus II, P = 0.521; group serum creatinine: DD versus ID, P = 0.073).

## Discussion

Over the last two decades, many studies have investigated the relationship between ACE I/D gene polymorphism and kidney transplantation to determine if gene polymorphisms have any influence over patient survival after transplantation. However, the results of these studies have been inconclusive. Therefore, we performed a meta-analysis to examine the association between ACE I/D polymorphism and renal transplantation based on clinical data from published studies.

In this study, 21 case–control studies with 1497 cases and 2029 controls were included. An additional ten studies that contained quantitative data on 814 patients were also included. The results provided strong evidence that ACE I/D polymorphism is associated with patient prognosis after kidney transplantation. This association is mainly evidenced in AR episodes and serum creatinine level. However, no objective evidence demonstrated that hypertension after transplantation is associated with ACE I/D polymorphism.

The DD+ID genotypes were risk factors for the occurrence of AR in the overall study population. Compared with patients carrying the II genotype, patients carrying the D allele showed higher risk for AR occurrence despite the lack of statistically significant differences. This result suggests that the D allele is a risk gene for AR. Considering that AR is an independent risk factor for graft loss [30], we can infer that recipients who carry DD+ID genes can incur graft loss in a short time. This phenomenon may be explained by the D allele increasing levels of ACE with enhanced conversion of angiotensin I to II [20]. Angiotensin II, the major effector of RAS, can participate in immunologically induced inflammation via nuclear factor-kappaB and upregulate TNF-α as well as pro-inflammatory mediators such as IL-6 and MCP-1 [31, 32]. TNF-α plays an important role in allograft AR by inducing the expression of histocompatibility antigens and by affecting the polarization of T cells towards Th1 profile [33]. As a consequence, recipients carrying the D allele have a higher chance of AR. A similar trend was found for CR but the results were not statistically significant, which may be attributed to small sample size. Stratified analysis revealed that Brazilian population have significantly higher risk for rejection among the five genetic models compared with Caucasians. This result indicates that population has significant influence on rejection.

Recipients with the DD genotype had extremely higher serum creatinine concentrations than those with the ID genotype. Furthermore, stratified analysis showed statistically significant differences between DD and ID genotype patients who had undergone transplantation less than a year. The results revealed that short-time graft function had larger contribution to this variance than long-time graft function. Despite the lack of significant difference between the DD versus II and ID versus II models, the DD genotype had higher serum creatinine level than the II and ID genotypes after kidney transplantation. Furthermore, different results were obtained in ethnic subgroup analyses for the three models, probably suggesting that ethnicity is an important influencing factor for allograft function. Several studies have reported that recipients with the DD genotype possess deteriorated renal transplant function or high chronic allograft dysfunction [3, 19, 22]. Fedor et al. [14] suggested that patients carrying the DD genotype are at high risk for developing chronic allograft nephropathy. This result can be attributed to the higher serum ACE concentration or serum ACE activity in patients carrying the DD genotype than in those carrying the other genotypes [14]. The high levels of ACE stimulating angiotensin II conversion [20] can also induce the production of angiotensin II-induced transforming growth factor-β (TGF-β). TGF-β expression may lead to the development of glomerulosclerosis and renal fibrosis [34]. In addition, angiotensin II exerts immunoregulatory effects, including leukocyte adhesion and chemotaxis [35]. These phenomena likely account for the poor graft function observed among DD genotype recipients. This suggests that when high serum creatinine concentrations are observed, early identification of the DD genotype must be achieved to focus on allograft functions of patients.

Cardiovascular complications are major obstacles in increasing survival after renal transplantation [36]. Hypertension, a common cardiovascular complication, can cause allograft dysfunction, organ damage and even death [37]. The level of blood pressure under different genetic models was analysed by comparing the number of patients with hypertension among the study groups. However, no statistically significant difference was found. This result suggested that ACE I/D polymorphism may not have any influence on hypertension among transplant patients.

In a genome-wide association study (GWAS), O'Brien, RP. et al. [38] examined long-term graft survival and allograft function in kidney transplant recipients. They identified two variants showing borderline genome-wide significance. Akcay et al. [22] have reported that ACE I/D polymorphism is also associated with outcome of allograft function after kidney transplantation. Unlike O'Brien’s study, our study focused only on ACE I/D polymorphisms reported in the literature. Besides, in addition to measuring serum creatinine levels, we also examined two other parameters, namely rejection and hypertension. Several limitations of the current study should be noted in interpreting our results. First, original data on the ACE genotypes are lacking. Therefore, existing data were used to analyse the influence of gene polymorphisms. Second, this study was restricted to Caucasian, Asian and Brazilian populations, with Caucasians comprising the majority sub-population. Third, eligible information was extracted from tabular data rather than individual data of every recipient, which may have contributed to inflation of standard errors in the pooled analyses. Lastly, genotype distribution among the controls did not show complete agreement with HWE. However, the only disagreement could be attributed to Chinese subjects included in the study.

In conclusion, ACE I/D polymorphism is associated with AR and allograft function after kidney transplantation. Early identification of ACE I/D genotype can significantly improve the outcome of patients. Well-designed and large-scale studies are necessary to further verify the present findings.

## Supporting Information

S1 Checklist PRISMA checklist. (DOC)

S2 Checklist Meta-analysis on genetic association studies checklist. (DOC)

S1 File PRISMA Flow Diagram. (DOC)

## References

[1] Kabat-Koperska J, Baskiewicz-Masiuk M, Safranow K, Golembiewska E, Paczkowska E, Miklaszewicz A, et al The influence of angiotensin-converting enzyme gene of donor and recipient on the function of transplanted kidney. Transplantation proceedings. 2005;37(2):755–9. 10.1016/j.transproceed.2004.12.175 . 15848522

[2] Filler G, Yang F, Martin A, Stolpe J, Neumayer HH, Hocher B. Renin angiotensin system gene polymorphisms in pediatric renal transplant recipients. Pediatric transplantation. 2001;5(3):166–73. . 11422818 10.1034/j.1399-3046.2001.00053.x

[3] Siekierka-Harreis M, Kuhr N, Willers R, Ivens K, Grabensee B, Mondry A, et al Impact of genetic polymorphisms of the renin-angiotensin system and of non-genetic factors on kidney transplant function—a single-center experience. Clinical transplantation. 2009;23(5):606–15. 10.1111/j.1399-0012.2009.01033.x . 19681973

[4] Sharma AM, Beige J, Distler A. Role of genetic variants of the renin-angiotensin system in chronic renal allograft injury. Kidney international. 1998;53(6):1461–5. 10.1046/j.1523-1755.1998.00930.x . 9607175

[5] Hostetter TH. Chronic transplant rejection. Kidney international. 1994;46(1):266–79. . 7933845 10.1038/ki.1994.269

[6] Solez K, Benediktsson H, Cavallo T, Croker B, Demetris AJ, Drachenberg C, et al Report of the Third Banff Conference on Allograft Pathology (July 20–24, 1995) on classification and lesion scoring in renal allograft pathology. Transplantation proceedings. 1996;28(1):441–4. . 8644308

[7] Ferrao FM, Lara LS, Lowe J. Renin-angiotensin system in the kidney: What is new? World journal of nephrology. 2014;3(3):64–76. 10.5527/wjn.v3.i3.64 25332897 PMC4202493

[8] Navar LG, Prieto MC, Satou R, Kobori H. Intrarenal angiotensin II and its contribution to the genesis of chronic hypertension. Current opinion in pharmacology. 2011;11(2):180–6. 10.1016/j.coph.2011.01.009 21339086 PMC3075356

[9] McDonough AA. Mechanisms of proximal tubule sodium transport regulation that link extracellular fluid volume and blood pressure. American journal of physiology Regulatory, integrative and comparative physiology. 2010;298(4):R851–61. 10.1152/ajpregu.00002.2010 20106993 PMC2853398

[10] Probstfield JL, O'Brien KD. Progression of cardiovascular damage: the role of renin-angiotensin system blockade. The American journal of cardiology. 2010;105(1 Suppl):10A–20A. 10.1016/j.amjcard.2009.10.006 . 20102969

[11] Rigat B, Hubert C, Alhenc-Gelas F, Cambien F, Corvol P, Soubrier F. An insertion/deletion polymorphism in the angiotensin I-converting enzyme gene accounting for half the variance of serum enzyme levels. The Journal of clinical investigation. 1990;86(4):1343–6. 10.1172/JCI114844 1976655 PMC296868

[12] Fellstrom B. Nonimmune risk factors for chronic renal allograft disfunction. Transplantation. 2001;71(11 Suppl):SS10–6. . 11583483

[13] Rieder MJ, Taylor SL, Clark AG, Nickerson DA. Sequence variation in the human angiotensin converting enzyme. Nature genetics. 1999;22(1):59–62. 10.1038/8760 . 10319862

[14] Fedor R, Asztalos L, Locsey L, Szabo L, Manyine IS, Fagyas M, et al Insertion/Deletion polymorphism of Angiotensin-converting enzyme as a risk factor for chronic allograft nephropathy. Transplantation proceedings. 2010;42(6):2304–8. 10.1016/j.transproceed.2010.05.020 . 20692468

[15] Cochran WG. The comparison of percentages in matched samples. Biometrika. 1950;37(3–4):256–66. . 14801052

[16] Egger M, Davey Smith G, Schneider M, Minder C. Bias in meta-analysis detected by a simple, graphical test. Bmj. 1997;315(7109):629–34. 9310563 10.1136/bmj.315.7109.629 PMC2127453

[17] Stuck AE, Rubenstein LZ, Wieland D. Bias in meta-analysis detected by a simple, graphical test. Asymmetry detected in funnel plot was probably due to true heterogeneity. Bmj. 1998;316(7129):469; author reply 70–1. 9492685 PMC2665578

[18] Beige J, Scherer S, Weber A, Engeli S, Offermann G, Opelz G, et al Angiotensin-converting enzyme genotype and renal allograft survival. Journal of the American Society of Nephrology: JASN. 1997;8(8):1319–23. . 9259361 10.1681/ASN.V881319

[19] Amorim CE, Nogueira E, Almeida SS, Gomes PP, Bacurau RF, Ozaki KS, et al Clinical impact of an angiotensin I-converting enzyme insertion/deletion and kinin B2 receptor +9/-9 polymorphisms in the prognosis of renal transplantation. Biological chemistry. 2013;394(3):369–77. 10.1515/hsz-2012-0314 . 23362199

[20] Broekroelofs J, Stegeman CA, Navis G, Tegzess AM, De Zeeuw D, De Jong PE. Risk factors for long-term renal survival after renal transplantation: a role for angiotensin-converting enzyme (insertion/deletion) polymorphism? Journal of the American Society of Nephrology: JASN. 1998;9(11):2075–81. . 9808093 10.1681/ASN.V9112075

[21] Viklicky O, Hubacek JA, Pitha J, Teplan V, Heemann UW, Lacha J, et al ACE gene polymorphism and long-term renal graft function. Clinical biochemistry. 2001;34(1):87–90. . 11239522 10.1016/s0009-9120(00)00202-2

[22] Akcay A, Sezer S, Ozdemir FN, Arat Z, Atac FB, Verdi H, et al Association of the genetic polymorphisms of the renin-angiotensin system and endothelial nitric oxide synthase with chronic renal transplant dysfunction. Transplantation. 2004;78(6):892–8. . 15385810 10.1097/01.tp.0000134972.81306.b1

[23] Zhang G, Wang H, Wang F, Yu L, Yang X, Meng J, et al Gene polymorphisms of the renin-angiotensin-aldosterone system and angiotensin II type 1-receptor activating antibodies in renal rejection. The Tohoku journal of experimental medicine. 2007;213(3):203–14. . 17984617 10.1620/tjem.213.203

[24] Yildiz A, Yazici H, Cine N, Akkaya V, Kayacan SM, Sever MS, et al The effect of angiotensin converting enzyme gene polymorphism on chronic allograft dysfunction in living donor renal transplant recipients. Clinical transplantation. 2002;16(3):173–9. . 12010139 10.1034/j.1399-0012.2002.01058.x

[25] Argani H, Noroozianavval M, Aghaeishahsavari M, Veisi P, Rashtchizadeh N, Ghorbanihaghjo A, et al Renin-angiotensin system polymorphisms and renal graft function in renal transplant recipients. Saudi medical journal. 2007;28(10):1496–502. . 17914507

[26] Beige J, Offermann G, Distler A, Sharma AM. Angiotensin-converting-enzyme insertion/deletion genotype and long-term renal allograft survival. Nephrology, dialysis, transplantation: official publication of the European Dialysis and Transplant Association—European Renal Association. 1998;13(3):735–8. . 9550656 10.1093/ndt/13.3.735

[27] Chudek J, Szotowska M, Karkoszka H, Verbeke F, Trautsolt W, Gumprecht J, et al Genotypes of renin-angiotensin system and plasma adiponectin concentration in kidney transplant patients. Annals of transplantation: quarterly of the Polish Transplantation Society. 2013;18:593–603. 10.12659/AOT.884022 . 24185422

[28] Hernandez D, Lacalzada J, Rufino M, Torres A, Martin N, Barragan A, et al Prediction of left ventricular mass changes after renal transplantation by polymorphism of the angiotensin-converting-enzyme gene. Kidney international. 1997;51(4):1205–11. . 9083287 10.1038/ki.1997.164

[29] Basset el EA, Berthoux P, Cecillon S, Deprle C, Thibaudin D, De Filippis JP, et al Hypertension after renal transplantation and polymorphism of genes involved in essential hypertension: ACE, AGT, AT1 R and ecNOS. Clinical nephrology. 2002;57(3):192–200. . 11926202 10.5414/cnp57192

[30] Kujawa-Szewieczek A, Kolonko A, Kocierz M, Szotowska M, Trusolt W, Karkoszka H, et al Association between gene polymorphisms of the components of the renin-angiotensin-aldosteron system, graft function, and the prevalence of hypertension, anemia, and erythrocytosis after kidney transplantation. Transplantation proceedings. 2011;43(8):2957–63. 10.1016/j.transproceed.2011.07.016 . 21996200

[31] Ruiz-Ortega M, Ruperez M, Lorenzo O, Esteban V, Blanco J, Mezzano S, et al Angiotensin II regulates the synthesis of proinflammatory cytokines and chemokines in the kidney. Kidney international Supplement. 2002;(82):S12–22. . 12410849 10.1046/j.1523-1755.62.s82.4.x

[32] Suzuki Y, Ruiz-Ortega M, Lorenzo O, Ruperez M, Esteban V, Egido J. Inflammation and angiotensin II. The international journal of biochemistry & cell biology. 2003;35(6):881–900. . 12676174 10.1016/s1357-2725(02)00271-6

[33] Zhao Z, Wang L, Yang C, Zhao T, Li L, Hu L, et al Soluble FGL2 induced by tumor necrosis factor-alpha and interferon-gamma in CD4+ T cells through MAPK pathway in human renal allograft acute rejection. The Journal of surgical research. 2013;184(2):1114–22. 10.1016/j.jss.2013.04.011 . 23664593

[34] De Albuquerque DA, Saxena V, Adams DE, Boivin GP, Brunner HI, Witte DP, et al An ACE inhibitor reduces Th2 cytokines and TGF-beta1 and TGF-beta2 isoforms in murine lupus nephritis. Kidney international. 2004;65(3):846–59. 10.1111/j.1523-1755.2004.00462.x 14871404 PMC2291513

[35] Szabo A, Lutz J, Schleimer K, Antus B, Hamar P, Philipp T, et al Effect of angiotensin-converting enzyme inhibition on growth factor mRNA in chronic renal allograft rejection in the rat. Kidney international. 2000;57(3):982–91. 10.1046/j.1523-1755.2000.00926.x . 10720951

[36] Fedor R, Asztalos L, Locsey L, Szabo L, Manyine IS, Fagyas M, et al Insertion/deletion polymorphism of the angiotensin-converting enzyme predicts left ventricular hypertrophy after renal transplantation. Transplantation proceedings. 2011;43(4):1259–60. 10.1016/j.transproceed.2011.03.064 . 21620105

[37] Serdaroglu E, Mir S, Berdeli A. Hypertension and ace gene insertion/deletion polymorphism in pediatric renal transplant patients. Pediatric transplantation. 2005;9(5):612–7. 10.1111/j.1399-3046.2005.00353.x . 16176418

[38] O'Brien RP, Phelan PJ, Conroy J, O'Kelly P, Green A, Keogan M, et al A genome-wide association study of recipient genotype and medium-term kidney allograft function. Clinical transplantation. 2013;27(3):379–87. 10.1111/ctr.12093 . 23432519

