# Supplementary material for: Association between Angiotensin I-Converting Enzyme Insertion/Deletion Polymorphism and Prognosis of Kidney Transplantation: A Meta-Analysis
Source: PLoS One. 2015 May 22;10(5):e0127320. doi: 10.1371/journal.pone.0127320 (PMC4441456; doi:10.1371/journal.pone.0127320)
Supplement: S1 File — (DOC) [file pone.0127320.s003.doc]

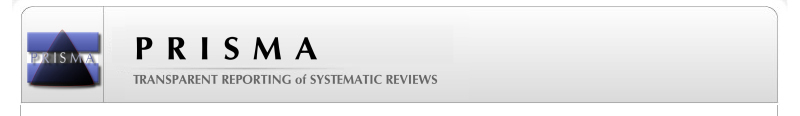
**PRISMA 2009 Flow Diagram**

**Screening**

**Included**

**Eligibility**

**Identification**

Records identified through database searching
(n = 83 )

Additional records identified through other sources
(n = 0 )

Records after duplicates removed
(n = 83 )

Records screened
(n = 40 )

Records excluded due to:

1) obviously irrelevant (n= 42)

2) not published in English (n= 1)

Full-text articles assessed for eligibility
(n = 13 )

Full-text articles excluded with reasons:

1) paediatric recipients (n= 4)

2) not comparing the clinical outcomes between different genotypes or wrong control group (n= 15)

3) information unavailable (n= 8)

Articles included in qualitative synthesis
(n = 13 )

Articles included in quantitative synthesis (meta-analysis)
(n = 13 )
